# Immunomodulatory Effect of *Rhaphidophora korthalsii* on Natural Killer Cell Cytotoxicity

**DOI:** 10.1155/2012/786487

**Published:** 2011-09-15

**Authors:** Swee Keong Yeap, Abdul Rahman Omar, Abdul Manaf Ali, Wan Yong Ho, Boon Kee Beh, Noorjahan Banu Alitheen

**Affiliations:** ^1^Institute of Bioscience, Putra University, Malaysia, Serdang, 43400 Selangor, Malaysia; ^2^Department of Veterinary Pathology and Microbiology, Faculty of Veterinary Medicine, Putra University, Malaysia, Serdang, 43400 Selangor, Malaysia; ^3^Faculty of Agriculture and Biotechnology, Sultan Zainal Abidin University, Malaysia (UniSZA), Kota Campus, Jalan Sultan Mahmud, 20400 Kuala Terengganu, Malaysia; ^4^Department of Cell and Molecular Biology, Faculty of Biotechnology and Biomolecular Sciences, Putra University, Malaysia, Serdang, 43400 Selangor, Malaysia; ^5^Department of Bioprocess Technology, Faculty of Biotechnology and Biomolecular Sciences, Putra University, Malaysia, Serdang, 43400 Selangor, Malaysia

## Abstract

The *in vivo* immunomodulatory effect of ethanolic extracts from leaves of *Rhaphidophora korthalsii* was determined via immune cell proliferation, T/NK cell phenotyping, and splenocyte cytotoxicity of BALB/c mice after 5 consecutive days of i.p. administration at various concentrations. Splenocyte proliferation index, cytotoxicity, peripheral blood T/NK cell population, and plasma cytokine (IL-2 and IFN-*γ*) in mice were assessed on day 5 and day 15. High concentration of extract (350 *μ*g/mice/day for 5 consecutive days) was able to stimulate immune cell proliferation, peripheral blood NK cell population, IL-2, and IFN- *γ* cytokines, as well as splenocyte cytotoxicity against Yac-1 cell line. Unlike rIL-2 which degraded rapidly, the stimulatory effect from the extract managed to last until day 15. These results suggested the potential of this extract as an alternative immunostimulator, and they encourage further study on guided fractionation and purification to identify the active ingredients that contribute to this *in vitro* and *in vivo* immunomodulatory activity.

## 1. Introduction

Since ancient times, the use of healing properties of plant extract has been common practice. Many people have started to use fresh plant products like fruits and vegetables in their daily meals and traditional herbs in different value-added processed forms (extracts, powders, pills, and decoction) as a daily food supplement to improve human health [1]. Among these, natural botanical sources that serve as potential immunostimulators have received much attention for their low toxicity and bioavailability [2]. Researchers have started to study the immunostimulatory effect of traditional herbs to supplement the traditional usage of these plants or herbs with a scientific basis. *Rhaphidophora korthalsii* (Araceae) is a large genus of a climbing shrub distributed throughout India, Sri Lanka, Cambodia, Venezuela, Malaysia, Australia, and Indonesia [3]. It is better known as dragon tail in Malaysia and Singapore and is commonly used in traditional Chinese herbal medicine for cancer and skin disease treatment [4]. Various extracts of *R. korthalsii *traditionally used for cancer treatment have been shown by researchers to exert cytotoxic effect on various cancerous cell lines [4, 5]. This effect was mainly driven by the presence of 5,6-dihydroxyindole (DHI) which showed cytotoxic activity against p388 and nonmelanocytic cancerous cell line [6]. Additionally, studies have also shown that an *R. korthalsii* methanol extract stimulated proliferation of mice splenocytes and human PBMC [4, 7]. As a result, they concluded that this plant extract may be useful in boosting the immune system to fight various diseases including cancer. However, the potential of the extract to stimulate *in vivo* immune function is still unclear. The present study aims to evaluate the *in vivo *effects of *R. korthalsii* on BALB/c mice in comparison with *in vitro* immunomodulatory effects of the extract in human PBMC.

## 2. Materials and Methods

### 2.1. Materials and Reagents

Recombinant murine interleukin 2 (rmIL-2; Dako, USA) was used as a positive control. This commercial immunomodulator was prepared by dissolving it directly into the culture media DMEM (Sigma, USA) at a concentration of 10,000 U/mL. Fluoroisothiocyanate- (FITC-) labeled CD3 antigoat monoclonal antibody and phycoerythrin- (PE-) labeled CD56 antigoat monoclonal antibody were purchased from Biolegend, USA.

### 2.2. Plant Material and Extraction

Leaves of *R. korthalsii* were collected from Georgetown, Penang in June 2006, and were identified by Mr. Lim Chung Lu (Kepong, Selangor) from the Forestry Division of the Forest Research Institute of Malaysia (FRIM, Malaysia). The voucher number of *R. korthalsii* is FRIM 33687. Leaves of the plant were air-dried in shade and finely powdered. Leaf extract was prepared by soaking the leaf powder in 250 mL of methanol (J.T. Baker, USA) for 72 hours. The extract was filtered with Whatman filter paper number 1 and evaporated to dryness under reduced pressure using Aspirator A-3S (EYELA, Japan) at <40°C. The process was repeated three times (yield 27.3%, w/w). The dried residue was resuspended in DMSO (Fisher Scientific, UK) at a concentration of 10 mg/mL as a plant extract stock. A substock solution of 0.2 mg/mL was prepared by diluting 20 *μ*L of the stock solution into 980 *μ*L serum-free culture medium (the percentage of DMSO in the experiment should not exceed 0.5). The stock and substock solutions were both stored at 4°C.

### 2.3. Animals

Balb/c mice, 8 weeks old, were used in this experiment. The animals were purchased from Animal House, Institute for Medical Research (IMR, Kuala Lumpur, Malaysia) and were housed under standard conditions at 25 ± 2°C, fed with standard pellets and tap water. This work was approved by Animal Care and Use Committee, Universiti Putra Malaysia (UPM; Ref: UPM/FPV/PS/3.2.1.551/AUP-R2).

### 2.4. *In Vivo* Stimulation of Mice with *R. korthalsii*

**Figure 1:**
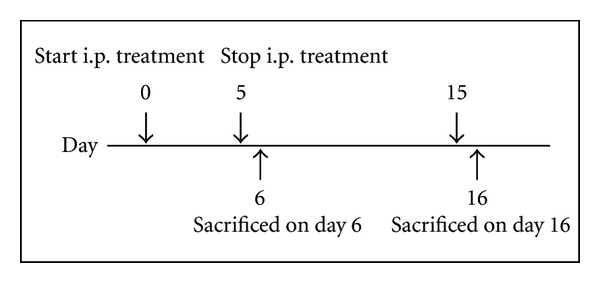
The general time course of the *in vivo* studies. The time of repletion and immune response assays varied somewhat among experiments. Animals were injected with extract, mouse rIL-2, or normal saline (for control group) for 5 days. Half of the animals (total of 30 mice) were sacrificed on days 6 and the remaining animals (the remaining 30 mice) were sacrificed on day 16.

Eight-week-old Balb/c mice ranging from 19 to 20 g were selected for this study, where duplicate treatment groups were conducted, but sacrificed on different days (D6 and D16). In each group, six sets of mice in groups of five were i.p. injected daily in the morning with either *R. korthalsii* extract (25, 200, 350, 700 *μ*g/mouse in 0.2 mL PBS), IL-2 (50 U in 0.2 mL PBS), or just 0.2 mL PBS for 5 days. Then, on day 6 of the study, the first group of animals was sacrificed while the second group was observed for 10 days and sacrificed on day 16 (Figure 1). Mice were anesthetised with 2% isoflurane (Merck) and sacrificed by cervical dislocation. Thus, all together, 60 mice were used in this study.

### 2.5. *Ex Vivo* Stimulation in the Proliferation Response of Spleen Cells

For *ex vivo* proliferation experiments, splenocytes and bone marrow monocyte from both day 6 and day 16 mice injected with *R. korthalsii* methanol extract were collected. Briefly, the spleen was removed and quickly washed with Hank's Balanced Salts Solution (HBSS), minced, and pressed through 80 *μ*m sterile wire mesh screen with a rubber syringe plunger. Added to this, femur was removed from mice, epiphysis was cut, and the bone marrow was triturated using an 18-gauge needle with HBSS and passed through 80 *μ*m sterile wire mesh. Both types of cell suspension were washed once with PBS supplemented with 0.1% BSA and 2 mg/mL EDTA (PBS-BSA-EDTA) and spun down at 200 g for 10 minutes. After that, red blood cells were removed by incubating and washing with lysis buffer (8 g NH_4_Cl, 1 g Na_2_EDTA, 0.1 g KH_2_PO_4_, pH 7.4). This step was repeated until the pellet was clean. Then, the cell suspension was washed a final time with PBS-BSA-EDTA, spun, the supernatant discarded, and the cell pellet was resuspended in 4 mL of Dulbecco's Modified Eagle Medium (DMEM) with 10% heat inactivated FBS. Cell counting was then performed to determine the lymphocyte cell number in the suspension. All of the steps above were carried out under sterile conditions in biological safety cabinet to prevent any contamination. Splenocyte cell viability for all treatment groups was determined after stimulation with *R. korthalsii* extract (25 *μ*g/mL), IL-2 (50 U/mL), or vehicle control and further incubation at 37°C, 5% CO_2_ for 72 hours. After this period, cells were harvested and cell growth was tested using MTT cell viability assay in triplicate in 96-well flat bottom plates. Briefly, 20 *μ*L of MTT (Sigma, USA) at 5 mg/mL was added into every well and the plate was incubated for another four hours at 37°C. Then, the plate was centrifuged at 200 ×g for 5 min, and 170 *μ*L of supernatant was aspirated from every well. The resulting formazan crystals were solubilized by 100 *μ*L of DMSO (Fisher Scientific, UK) in each well followed by incubation for 20 minutes at 37°C. Finally, the plate was read at 570 nm and 630 nm as reference wavelength by using *μ* Quant ELISA Reader (Bio-tek Instruments, USA). The percentage of cell viability was calculated using the following formula:

(1) $$\text{Percentage}\;\text{of}\;\text{cell}\;\text{viability}=\left(\frac{\text{OD}\;\text{sample}}{\text{OD}\;\text{control}}\right)\times 100.$$

### 2.6. *In Vivo* Mice Blood NK Cell Immunophenotyping

Blood was collected in heparinized tuberculin syringes via cardiac puncture and was washed three times with PBS-BSA-EDTA. Fifty *μ*L of the blood was resuspended with 500 *μ*L of lysing buffer (BD, USA) and 10 *μ*g/10 *μ*L FITC conjugated antimouse CD3 (17A2; isotype control: IgG2b, *κ*; Biolegend, USA) and 12.5 *μ*g/10 *μ*L PE conjugated antimouse NK-1.1 antibodies (PK136; isotype control: IgG2a, *κ*; Biolegend, USA). After that, cells were washed three times (300 g, 10 minutes) and resuspended in PBS-BSA-EDTA at a final volume of 1 mL for flowcytometric analysis using a FACSCalibur flowcytometer with CellQuest Pro software (BD Bioscience, USA).

### 2.7. *In Vivo* Mice Blood IL-2 and IFN-*γ* Determination

Blood was collected using cardiac puncture, placed into heparin tubes, and spun down at 8000 g for 5 minutes. The translucent blood plasmas was collected from the upper layer and stored at −20°C until future analysis. The concentration of IL-2 and IFN-*γ* in plasma was determined by using murine Cytokine Instant Enzyme Link Immunosorbent Assay (ELISA) kit (BioLegend, USA). Briefly, capturing antibody specific to murine IL-2 or IFN-*γ* was coated onto the wells of the microtitre strips provided one day before the assay at 4°C. Then, 100 *μ*L of diluted IL-2 or IFN-*γ* standard (range from 125 *μ*g to 2 *μ*g) and plasma were added into the precoated microtitre plate and incubated for 2 hours at room temperature. The plates were then washed and 100 *μ*L of diluted biotinylated detection antibody was added and incubated for 2 h. Imaging step included Avidin-Horse Radish peroxidase and 3,3′,5,5′ tetramethylbenzidine (TMB) as chromogen. OD was read immediately at 450 nm wavelength using *μ* Quant ELISA Reader (Bio-Tek Instruments, USA) at Animal Tissue Culture Laboratory, FBBS, UPM. The result was compared to the control prepared simultaneously. Each plasma from either treated or nontreated animals was assayed three times. Data are expressed as pg/mL.

### 2.8. *In Vivo* Induction of Mice Splenocytes Cytotoxicity

Splenocytes (Effector-E) were harvested from the control, IL-2, and extract treated animals, and the cytotoxicity of splenocytes towards Yac-1 (Target-T) cell line was determined using CytoTox 96 nonradioactive cytotoxicity assay kit (Promega, USA) at the ratio of effector : target of 2 : 1; 10 : 1, and 50 : 1 and incubated in 37°C, 5% CO_2_, and 90% humidity for 24 hours. Splenocyte with the cell concentration equivalent to each ratio in the coculture experimental well (effector spontaneous LDH release) and Yac-1 with the cell concentration equivalent to the coculture experimental well (for both target spontaneous LDH release and target maximum LDH release by the lysis solution) were prepared simultaneously. Briefly, 45 minutes prior to the ending of coculture incubation period, 10 *μ*L of Triton-X lysis solution (10 X) was added into the target Yac-1 cell maximum LDH release control wells. The plate was further incubated for 45 minutes until the target cells maximum LDH release control was completely lysed. Then, the plate was centrifuged (250 g, 4 minutes), and 50 *μ*L of supernatant from each well was transferred to a new 96-well plate (BD Biosciences, USA). The substrate mixture was prepared simultaneously by reconstituting one vial of substrate mixing with 12 mL of assay buffer. After that, 50 *μ*L of reconstituted substrate mixture was added into all the experimental wells and incubated for 30 minutes in the dark. Finally, 50 *μ*L of 2.5 N sulfuric acid stop solution was added into each well and the plate was read at 490 nm wavelength immediately by using *μ* Quant ELISA Reader (Bio-Tek Instruments, USA). LDH converts substrate mixture into a yellow formazan product. The intensity of color formed (recorded as absorbance at 496 nm) is proportional to the number of lysed cells. Percentage of LDH release was calculated using the following formula:

(2) $$\begin{array}{cc} & \text{Percentage}\;\text{of}\;\text{Cytotoxicity}\\ & \;=\frac{\text{OD}\;\text{sample}-\text{OD}\;\text{target}\;\text{spontaneous}}{\text{OD}\;\text{target}\;\text{maximum}-\text{OD}\;\text{target}\;\text{spontaneous}}\\ & \;\;-\frac{\text{OD}\;\text{effector}\;\text{spontaneous}}{\text{OD}\;\text{target}\;\text{maximum}-\text{OD}\;\text{target}\;\text{spontaneous}}\\ & \;\;\times 100. \end{array}$$

### 2.9. Statistical Analysis

Results are expressed as Mean ± Standard Error (SEM). Differences between means were evaluated using ANOVA test (one way) followed by Duncan test, and *P* ≤ 0.05 was taken as statistically significant.

## 3. Results

### 3.1. *In Vivo* Stimulation of Mice

In view of the significant *in vitro* stimulatory activity by *R. korthalsii *[4, 7], it was of interest to determine if this activity would translate into an *in vivo* effect. In order to assess the *in vivo* immunoregulatory effect of *R. korthalsii* methanol extract, 60 mice weighing approximately 19-20 g were divided into 12 groups and were injected with different concentration of extract or positive control. Mice weighed between 23 to 25 g after day 5 of injection and 28 to 30 g after day 15 of first injection. None of the animals died during the experimental period, and there were no differences in activity between the untreated and treated mice.

### 3.2. *Ex Vivo* Splenocyte and Bone Marrow Proliferation

The *ex vivo* proliferation assay is an *in vitro* assessment which allows the study of *in vivo* splenocyte proliferation after being treated with the immunomodulator [8]. The *in vitro *proliferation response of splenocytes and bone marrow cultures was investigated 72 hours after harvesting from animals by treatment with test extract, positive, and negative controls. After 5 days of *in vivo* treatment with the 2 high concentrations of extract (350 and 700 *μ*g/mice) or rIL-2, *ex vivo* stimulation with extract or rIL-2 greatly increased the proliferation response of splenocytes and bone marrow cells (Table 1). In contrast, low concentration of extract (25 and 200 *μ*g/mice) groups responded to *in vitro* extract and rIL-2 stimulation quite similar to the splenocytes and bone marrow cells isolated from the control group. This could be because the extract concentration is too low to initiate the indirect mitogenic effect towards the immune cells *in vivo*. Recombinant IL-2 was sufficient to induce cell growth in both splenocytes and bone marrow with or without the *ex vivo* stimulation. The mice injected with high concentration (350 and 700 *μ*g/mice) extract showed the greatest effect on splenocyte and bone marrow cells growth. *Ex vivo* stimulation on the high concentration extract treated mice exhibited marked increase of both splenocytes and bone marrow cell growth, even compared to the optimum stimulation (25 *μ*g/mL) effect on the same type of cell *in vitro *in a previous report [7]. After day 15, there was no major difference for the cell growth response when compared to day 5 in the high dose extract-treated mice. However, for the mice injected with rIL-2, there was a marked decrease of *ex vivo* cell growth in both splenocytes and bone marrow cell from day 6 to day 16. This may be due to the *in vivo* degradation of rIL-2 10 days after the injection. Thus, both the extract and rIL-2 were able to promote the immune cell growth not only *in vitro*, but also *in vivo* and *ex vivo*.

### 3.3. *In Vivo* Blood NK Cell Immunophenotyping

**Figure 2:**
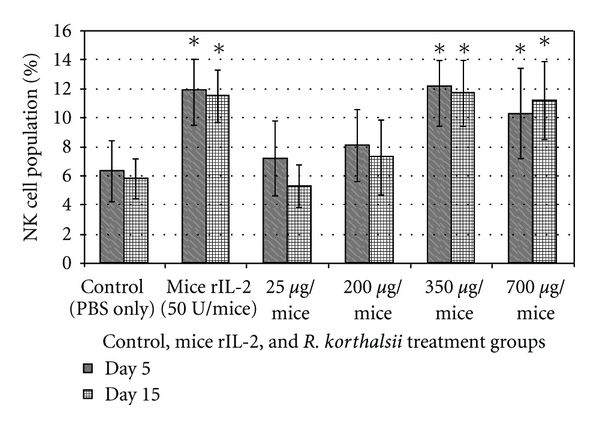
NK cell immunophenotyping on mice blood after being treated with various concentration of *R. korthalsii *methanol extract or mice rIL-2 for day 6 and day 16 *in vivo. *Each value represents the means ± SEM for three assays in duplicate each. The differences between the control group and treated group were determined by one-way ANOVA (*P ≤ 0.05).

In order to understand the *in vivo* stimulatory effect of the extract on NK cells population, NK cell immunophenotyping was performed (Figure 2). The peripheral blood of mice treated with extract or rIL-2 was found to increase the cell population with NK 1.1 phenotype as compared with nontreated control mice. The increase was found only by using rIL-2 and high concentrations (350 and 700 *μ*g/mice) of *R. korthalsii* methanol extract but not with the mice treated with lower concentration (25 and 200 *μ*g/mice) of extract. The increase in cell population with NK1.1 phenotype was maintained until day 15 without significant reduction.

### 3.4. *In Vivo* Blood Cytokine ELISA Test

**Figure 3:**
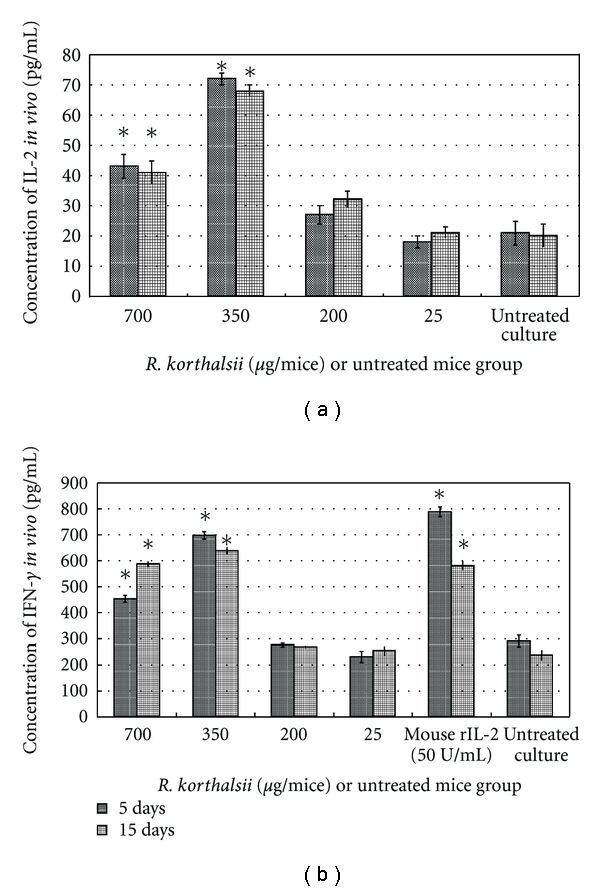
Effects of *R. korthalsii* methanol extract on cytokine production *in vivo*. The serum concentrations of IL-2 and IFN-*γ* were tested by ELISA assay. (a) Concentration of IL-2 *in vivo*. (b) Concentration of IFN-*γin vivo*. Each value represents the means ± SEM for three assays in duplicate each. The differences between the control group and treated group were determined by one-way ANOVA (*P ≤ 0.05).

*In vivo* study results were in concordance with the *in vitro* study where both extract and mouse rIL-2 were able to stimulate the production of cytokines.  Figure 3 illustrates the cumulative amount of rIL-2 and IFN-*γ* in the blood after the treatment. Mouse rIL-2 was not used as positive control in the rIL-2 ELISA test to avoid false positives given by the injection. Mice treated with 700 and 350 *μ*g/mice of extract showed approximately 2- to 3.5-fold higher levels of IL-2 and IFN-*γ* detected in blood plasma. On the other hand, the low concentration (25 and 200 *μ*g/mice) of extract did not show significant differences for either cytokines detected in blood plasma as compared to untreated mice at day 5 or 15 mice. Mouse rIL-2 treatment produced the highest level of IFN-*γ* detected in blood plasma as compared to all concentration of extract. However, both treatments also induced lower level of cytokines detected in blood plasma when compared to the *in vitro* splenocytes cytokines production. Day 5 and day 15 contained similar a level of cytokines detected in the blood plasma and gave the idea that both treatment lasted from day 5 until day 15.

### 3.5. *In Vivo* Mice Splenocytes Cytotoxicity Assay

**Figure 4:**
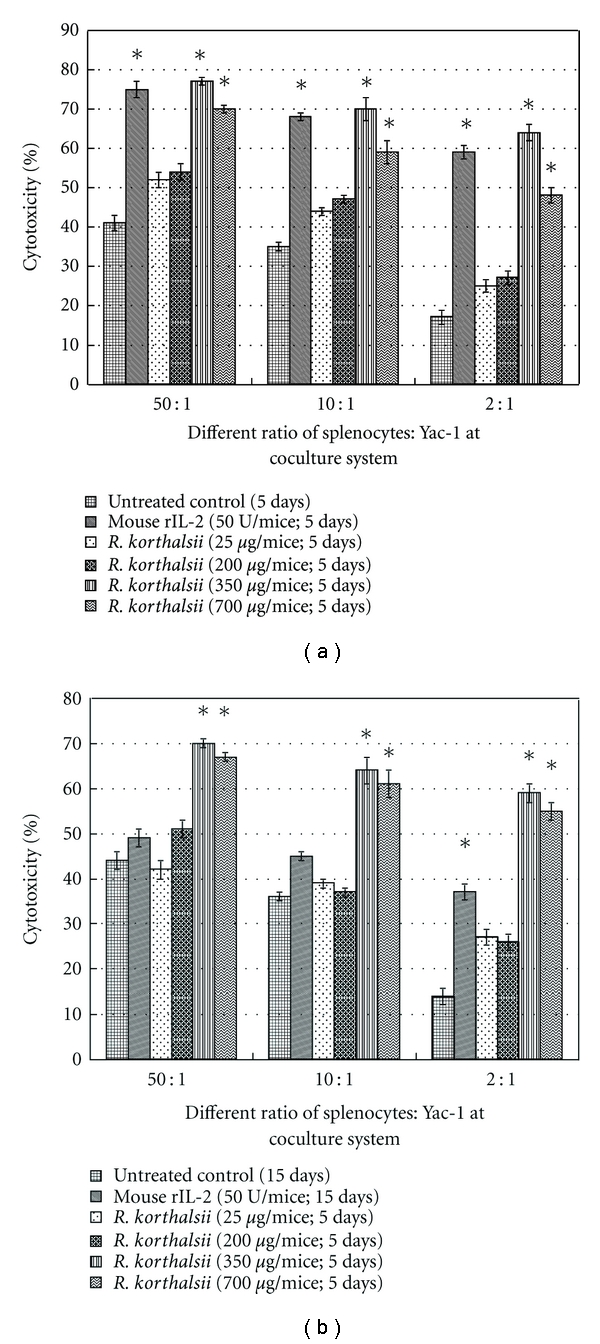
Viability of Yac-1 after being treated with different ratio of *R. korthalsii* extract or mouse rhIL-2-activated splenocytes *in vivo* (a) after day 5 of treatment and (b) after day 15 of treatment. Each value represents the means ± SEM for three assays in triplicate each. The differences between the control group and treated group were determined by one-way ANOVA (*P ≤ 0.05).

The result of *in vivo* induction of splenocyte activity by extract and rIL-2 is shown in Figure 4. Cytotoxicity of untreated and low concentration (25 and 200 *μ*g/mice) extract treated splenocytes were quite similar where we measured an increase from approximately 20% to 40–50% cytotoxicity when increasing the splenocyte cell number in the coculture. This result was similar on both day 6 and day 16 mice. On the other hand, mice treated with a high concentration of extract demonstrated an enhanced splenocyte cytotoxicity, even at low ratio of effector cells at both time points. Although the rIL-2 showed a similar effect on splenocyte cytotoxicity when compared to high concentrations of *R. korthalsii* methanol extract, it was short term since the effect in day 16 mice dropped to a level same as the control group while the extract still exhibited a strong stimulative effect on splenocyte cytotoxicity. From this result, *R. korthlasii* methanol extract was found to enhance the splenocytes cytotoxicity *in vivo* and was longer lasting when compared to rIL-2.

## 4. Discussion

Searching for substances with immunostimulative or immunorestorative effects could contribute to the maintenance of the immune system, which may prevent the progression of tumour establishment [9]. Evaluation of immune cells, especially bone marrow cells and splenocyte proliferation, is typical when screening for potential immunomodulatory effects of a substance [10].

*In vivo *study using small animals to substitute for human trial can provide a better understanding of the immunomodulator toward the immune system as compared to the single-cell type *in vitro *studies [11–13]. For example, Kagi et al. [14] showed the importance of the pore-forming protein-perforin for *in vivo* antitumour effects using perforin-deficient mice. Besides, animal experiments have also been used to demonstrate the ability of raw herbs or some of their purified components to inhibit tumour growth through activation of the immune system [15, 16] or suppress the immune system [17]. 

Since previous study showed that continuous IL-2 administration was more effective to prolong augmentation of NK cell competence *in vivo*, we selected 5 days of repeated injections of *R. korthalsii* methanol extract or IL-2 treatment for the *in vivo *portion of our study [18]. Various concentrations of extract were evaluated, and results showed that low concentrations (25 and 200 *μ*g/mice) were insufficient to induce statistically significant immunomodulatory effects *in vivo*. On the other hand, higher concentrations (350 and 700 *μ*g/mice) showed significant immunostimulatory effects, as reported in the previous *in vitro* study by Yeap et al. [7]. The results of this study indicated the optimum concentration was 350 *μ*g/mouse/day, as the immunomodulatory effects in the 700 *μ*g/mouse/day group were slightly reduced in comparison.

From the *ex vivo* proliferation results, restimulation of the spleen cells with either extract or cytokine further enhanced the effect by both extract and rIL-2. This may be due to the “priming” effect brought about by the i.p. injections of the extract or cytokine. In the *ex vivo* bone marrow cells, higher growth rates were recorded after the 72 hours stimulation when compared to the *in vitro* cell viability results. This result may also be due to the contribution of “priming” effect of the extract or rIL-2 injection. Added to this, activation of NK cells may also contribute to better stimulation of bone marrow cell growth. NK cells have long been thought to regulate hematopoiesis [19]. Activation of NK cells with rIL-2 was found to support hematopoietic growth of bone marrow cells without any hematopoietic growth factors [20]. The *in vitro* cell viability study only involves the single-cell type with extract and does not account for the interaction of activated NK cells to stimulate bone marrow cell growth. Unlike the *in vitro* study, the *ex vivo* study involves more elements of the immune system with the presence of extract or rIL-2-activated NK cells, which may also further help to promote the maturation and proliferation of the hematopoietic cells *in vivo*. 

*In vivo* splenic cytotoxic NK cells' activity was evaluated by cocultivation of isolated splenocytes with the NK cell sensitive Yac-1 target. The results showed that the *in vivo* mouse model for immune cell cytotoxicity to target Yac-1 cells was dose dependent and statistically significant with respect to the control in the groups treated with extract. The results obtained in the present study showed that both treatment periods displayed a dose-dependent immunostimulatory effect which can last until day 15. However, rIL-2 was less durable as compared to the extract at high concentration. This may due to the rapid clearance of the protein from the systemic circulation following administration to the mice [21]. 

Previous studies [4, 7, 22] have reported on the stimulation of *in vitro *human PBMC and mice splenocytes cell by *R. korthalsii *methanol extract. Similar patent of stimulation of immune cell proliferation and cytokines (IL-2 and IFN-*γ*) secretion have been discovered in this *in vivo *study. IL-2 and IFN-*γ* is a type of cytokine that plays an important role to inhibit tumor formation. IL-2 was able to stimulate NK cell while IFN-*γ* was secreted by the activated NK cell [7, 23]. Thus, the potential of *R. korthalsii* methanol extract on stimulation of immune cell IL-2 secretion may be the major contributor of raise of IFN-*γ* producing NK cell population in the peripheral blood. Extract-activated NK cells may further contribute to the suppression of tumor cell lines (Yac-1, HepG2, and K562) as reported in the above results or the previous studies [7, 22]. 

In this study, stimulation of the immune system by *R. korthalsii* methanol extract had significant effects on proliferation, NK cell population, and cytokine expression in the host, which further enhanced the cytolytic activity of the immune cells. Up till now, 5,6-dihydroxyindole (DHI) is the only compound successfully isolated from *R. korthalsii*. However, role of DHI on the immunomodulatory effect of *R. korthalsii *methanol extract and the other potential active secondary metabolites that play an important role in this immunostimulatory effect are still unknown. Thus, future study should focus on isolation and evaluation of active metabolites from the extract that contributes to the immunomodulatory effect against the NK cell.

## Figures and Tables

**Table 1:** Mice splenocytes harvested after day 5 or day 15 of treatment were allowed to proliferate with or without the treatment of *R. korthalsii *(25 *μ*g/mL) or mouse rIL-2 (50 U/mL). The rate of cell viability was evaluated using MTT cell viability assay*.

	Untreated		*R. korthalsii *(25 *μ*g/mL)		Mouse rIL-2 (50 U/mL)	
	Splenocytes (%)	Bone marrow (%)	Splenocytes (%)	Bone marrow (%)	Splenocytes (%)	Bone marrow (%)

Control						
5 day	100	100	172 ± 1.3	152 ± 2.6	186 ± 2.1	160 ± 3.2
15 day	100	100	168 ± 2.1	154 ± 2.3	179 ± 3.4	159 ± 2.1
*R. korthalsii *(25 *μ*g/mice)						
5 day	104 ± 1.6	106 ± 1.3	178 ± 2.7	147 ± 1.4	184 ± 1.6	158 ± 2.7
15 day	102 ± 1.1	107 ± 2.5	174 ± 1.5	149 ± 1.9	187 ± 1.8	162 ± 2.8
*R. korthalsii *(200 *μ*g/mice)						
5 day	113 ± 1.2	107 ± 2.4	184 ± 3.2	159 ± 2.7	192 ± 3.4	163 ± 1.8
15 day	118 ± 1.6	110 ± 2.5	180 ± 2.7	151 ± 1.7	195 ± 2.3	171 ± 1.9
*R. korthalsii *(350 *μ*g/mice)						
5 day	154 ± 1.8*	161 ± 2.8*	247 ± 2.9*	201 ± 4.8*	253 ± 4.1*	214 ± 3.7*
15 day	151 ± 2.1	168 ± 3.4	241 ± 3.2	213 ± 3.6	258 ± 4.7	209 ± 4.2
*R. korthalsii *(700 *μ*g/mice)						
5 day	146 ± 2.3*	137 ± 2.4*	225 ± 3.4*	187 ± 3.1*	241 ± 3.5*	193 ± 3.1*
15 day	142 ± 1.5	141 ± 2.3	233 ± 4.6	192 ± 4.2	245 ± 3.7	204 ± 2.5
Mice rIL-2 (50 U/mice)						
5 day	143 ± 2.1*	172 ± 4.3*	259 ± 4.2*	211 ± 4.9*	267 ± 3.3*	219 ± 1.8*
15 day	124 ± 2.7	121 ± 2.6	197 ± 3.5	184 ± 5.1	207 ± 3.5	192 ± 1.9

*Note: the values are the means ± SE of three experiments. The differences between the control group and treated group were determined by one-way ANOVA (*P ≤ 0.05).
